# Epithelial Dysfunction in Congenital Diaphragmatic Hernia: Mechanisms, Models and Emerging Therapies

**DOI:** 10.3390/cells14100687

**Published:** 2025-05-09

**Authors:** Ophelia Aubert, Olivia M. Dinwoodie, Richard Wagner, Xingbin Ai

**Affiliations:** 1 Department of Pediatric Surgery, University Medical Center Mannheim, 68165 Mannheim, Germany; 2 Division of Newborn Medicine, Department of Pediatrics, Massachusetts General Hospital, Harvard Medical School, Boston, MA 02114, USA; odinwoodie@mgh.harvard.edu; 3 Department of Pediatric Surgery, University Hospital Leipzig, 04103 Leipzig, Germany; richard.wagner@medizin.uni-leipzig.de

**Keywords:** congenital diaphragmatic hernia, lung compression, airway epithelium, mechanotransduction, YAP, NFkB

## Abstract

Congenital diaphragmatic hernia (CDH) is a complex disorder whereby improper formation of the diaphragm allows herniation of the internal organs into the thoracic cavity, resulting in pulmonary hypoplasia among other complications. Although epithelial dysfunction is central to CDH pathology, relatively little attention has been paid to the underlying mechanisms orchestrating epithelial malfunction. Proinflammatory signaling downstream of impaired mechanotransduction due to in utero lung compression has been elucidated to drive epithelial cell phenotypes. This has been illustrated by a reduction in nuclear YAP and the upregulation of NF-kB in CDH models. In this review, we draw from recent findings using emerging technologies to examine epithelial cell mechanisms in CDH and discuss the role of compression as a central and, crucially, sufficient driver of CDH phenotypes. In recognition of the limitations of using genetic knockout models to recapitulate such a heterogenic and etiologically complicated disease, we discuss alternative models such as the established nitrofen rat model, air–liquid interface (ALI) cultures, organoids and ex vivo lung explants. Throughout, we acknowledge the importance of involving mechanical compression in the modeling of CDH in order to faithfully recapitulate the disease. Finally, we explore novel therapeutic strategies from stem cell and regenerative therapies to precision medicine and the importance of defining CDH endotypes in order to guide treatments.

## 1. Introduction

The lung epithelium is a heterogeneous tissue composed of specialized cell types, including alveolar type I and II cells, basal cells, club cells and ciliated cells [1,2]. Each population fulfills a distinct role contributing to gas exchange, surfactant production, mucociliary clearance and epithelial regeneration. The precise balance and spatial organization of these subsets are essential for normal lung development and postnatal function. In congenital diaphragmatic hernia (CDH), this epithelial diversity is disrupted. CDH lungs exhibit impaired epithelial differentiation, changes in abundance of key epithelial populations, as well as alterations in extracellular matrix composition and tissue architecture, collectively contributing to the hypoplastic phenotype [3,4,5,6,7].

**Figure 1:**
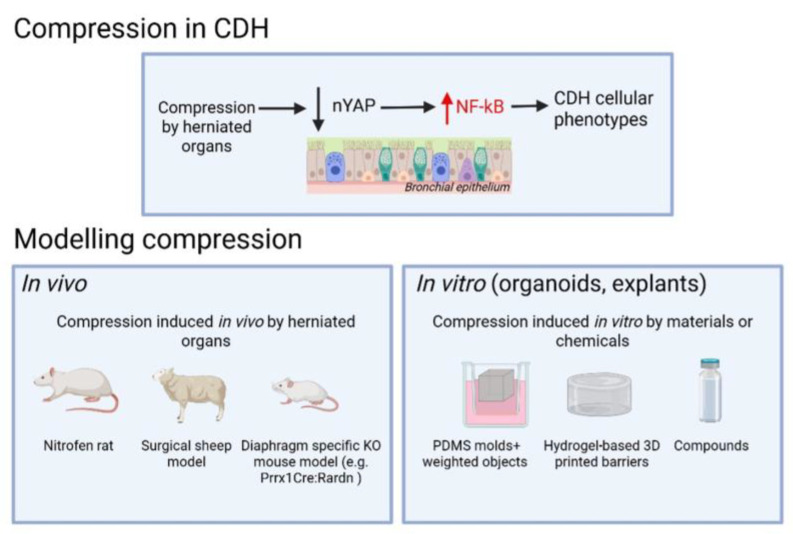
Schematic overview illustrating compression-induced CDH phenotypes and their experimental modeling using in vivo and in vitro methods.

Historically, CDH research has focused primarily on genetic, mesenchymal, vascular and diaphragmatic abnormalities [8,9]. In contrast, epithelial dysfunction—despite being central to respiratory failure in CDH—has received comparatively little attention. Recent evidence highlights epithelial immaturity and differentiation defects as key features of CDH-associated lung hypoplasia [3,4,5,10]. Proinflammatory signaling has emerged as a critical regulator of epithelial cell fate, and growing evidence suggests that impaired mechanotransduction—resulting from in utero lung compression—acts upstream of this inflammatory response [3,4,5,10,11]. Altered mechanical cues dysregulate YAP activity, triggering NF-κB signaling and establishing a proinflammatory environment that further impairs epithelial differentiation [4,11,12,13] (Figure 1). The importance of compression in inducing CDH lung phenotypes, therefore, must be considered when designing CDH models.

Advances in single-cell and spatial transcriptomics have provided high-resolution maps of lung cell states and their spatial organization in both normal and CDH lungs [10,14,15,16]. These technologies have revealed cell-type-specific transcriptional alterations and implicated dysregulated immune cells in CDH pathogenesis, offering key insights into the molecular consequences of disrupted mechanotransduction and inflammation. Although numerous genetic knockout mouse models have provided insight into lung development, many rely on homozygous mutations that fail to capture the heterogeneity and mostly sporadic nature of human CDH [17,18,19]. While genetic factors likely contribute to lung defects in a subset of CDH cases, this review focuses on the shared pathogenic event in CDH, lung compression by the herniated visceral organs, and aims to highlight emerging concepts and research tools with broader translational relevance (Figure 1).

In this review, we first outline recent discoveries and technologies that have refined our understanding of airway epithelial heterogeneity and lung dysfunction in CDH. We then discuss experimental systems—including air–liquid interface models, organoids, lung explants and in vivo models—and their respective abilities to recapitulate the lung phenotype in CDH. Finally, we explore therapeutic strategies targeting lung hypoplasia and differentiation defects.

## 2. Emerging Technologies and Concepts Unravel Lung Cellular Heterogeneity in CDH

### 2.1. Single-Cell RNA Sequencing and Spatial Transcriptomics

The hypoplastic lung in CDH exhibits significant cellular heterogeneity, which is increasingly being elucidated through technologies such as single-cell RNA sequencing (scRNA-seq). This high-resolution approach allows for detailed characterization of individual lung cells, uncovering disruptions in progenitor, endothelial, epithelial and immune cell populations in CDH. In a recent study [14], scRNA-seq analysis of CDH-derived lung organoids compared to healthy controls identified distinct disease-specific transcriptomic profiles, including an increased representation of alveolar type II (AT2) and neuroendocrine cells, alongside a reduction in basal and club cells, consistent with previous findings from our group [4,5,11]. Gene ontology analysis revealed that pre-fetoscopic endoluminal tracheal occlusion (FETO), CDH organoids exhibited upregulated pathways related to surfactant production and metabolism, including an increased expression of *SPC*, along with dysregulated extracellular matrix interactions [14]. Following FETO, RNA sequencing demonstrated a shift toward a more normalized gene expression profile, with the downregulation of extracellular matrix (ECM)-related pathways and the upregulation of phosphatidylcholine metabolism, suggesting improved tissue organization and epithelial maturation. The authors propose that associating transcriptomic alterations in CDH organoids with clinical outcomes could provide a valuable prenatal prognostic tool, potentially informing individualized therapeutic strategies.

Beyond human lung-derived organoid models, single-cell studies have also been applied to the nitrofen rat model of CDH [10,15]. This approach expands the scope of investigation beyond epithelial populations and into vascular and immune cell compartments. ScRNA-seq of CDH rat lungs revealed significant alterations in microvascular endothelial cells (mvECs), distinguishing CDH-specific changes from those induced by nitrofen exposure alone [15]. CDH mvECs exhibited a unique inflammatory transcriptomic profile, with upregulated genes linked to immune cell adhesion and oxidative stress. Conversely, genes essential for vascular function, including *Ca4*, *Apln* and *Ednrb*, were significantly downregulated, indicating impaired angiogenesis and vascular integrity. MvCa4^+^ endothelial cells, crucial for gas exchange and alveolar repair, were markedly reduced in CDH lungs, highlighting endothelial dysfunction as a potentially key contributor to pulmonary hypoplasia. In line with this, single-nucleus RNA sequencing revealed that fetal CDH rat lungs exhibit a multilineage inflammatory signature, characterized by macrophage enrichment and the upregulation of proinflammatory genes, suggesting a prenatal immune dysregulation that could further impair lung development [10].

Other omics approaches, such as spatial transcriptomics, complement scRNA-seq by preserving tissue architecture while enabling high-resolution mapping of gene expression across lung regions, uncovering spatially distinct alterations in CDH [16]. Analysis of CDH lungs demonstrated impaired angiogenesis, with the downregulation of *EPAS1* and *FHL1*, genes critical for endothelial function and vascular development. The upregulation of genes associated with inflammation, including the TNF-alpha and NF-kB signaling pathways, indicates an inflammatory microenvironment, consistent with previous findings [4,5,10,11]. The increased expression of macrophage marker CD163 further supports macrophage enrichment and immune dysregulation in CDH lungs. Additionally, genes involved in ECM remodeling (e.g., *COL1A1*) were upregulated, suggesting altered lung architecture and fibrotic remodeling. Vitamin A-responsive pathways, crucial for lung development, were enriched in control lungs but deficient in CDH lungs, underscoring the previously described role of retinoic acid signaling in pulmonary maturation and CDH pathogenesis [20,21,22]. However, these findings are subject to limitations, as spatial transcriptomic analysis can only be performed on lung tissue from affected neonates postnatally, introducing potential confounders from intensive care treatments.

Though not directly investigating lung hypoplasia, the use of scRNA-sequencing of the developing diaphragm in CDH has enabled the discovery of genetic abnormalities affecting diaphragm development in CDH, eventually resulting in lung hypoplasia among CDH phenotypes [23]. Data from this sequencing has allowed for the recent creation of diaphragm-specific knockout mouse models, such as the *Prrx1Cre:Rardn* mice, which lack the retinoic acid signaling receptor (Rardn) in the mesenchyme of the developing diaphragm (Prrx^+^ cells) and exhibit lung hypoplasia [23]. Focusing on diaphragm defects to create CDH models with pulmonary hypoplasia as a result of compression has been undertaken by other groups, and the use of these models represents a promising direction for future research [24,25].

### 2.2. Proteomic and Metabolomic Profiling

Different proteomic analyses have recently been used to identify protein expression patterns associated with CDH. For instance, Wagner et al. conducted a comprehensive untargeted proteomic profiling (LS-MS/MS) of hypoplastic lungs in the nitrofen CDH model (canalicular stage, E21), revealing >200 altered proteins related to an underlying inflammatory response as a key factor in abnormal lung development [26]. Their findings suggest that inflammation plays a pivotal role in the pathogenesis of lung hypoplasia observed in CDH patients. Another study on the nitrofen rat model at the pseudoglandular stage using tandem mass tag (TMT) proteomics identified 79 differentially expressed proteins between fetal CDH and control lungs. The altered proteins were linked to tight junction pathways (Cldn3, Magi1, Myh9), phospholipase D pathways and HIF-1 signaling [27]. Additionally, proteomic analysis of tracheal fluids in the CDH lamb model with FETO found that CDH suppressed, while tracheal occlusion promoted, cell proliferation and AKT-related signaling cascades [28]. Another study identified proteomic changes in human amniotic fluid of CDH and control cases [29]. Among 1036 proteins, 218 differed between CDH and controls, affecting GP6 signaling, MSP-RON signaling and cardiovascular development. Key proteins, including surfactant protein B, osteopontin, kallikrein 5 and galectin-3, were validated via ELISA, showing potential for CDH diagnosis and management. A report by Tachi et al. analyzed serum profiles in neonates with CDH using umbilical cord serum from isolated CDH cases (n = 4) and matched controls (n = 4) [30]. Liquid chromatography–tandem mass spectrometry detected 697 proteins, with 98 differentially expressed. Complement C1q showed the highest fold change, followed by complement C5. Pathway analysis revealed significant enrichment in complement and coagulation cascades, suggesting a potential role of the complement pathway in CDH pathophysiology.

Complementing proteomic data, metabolomics reveals pathway alterations relevant to CDH pathology and has progressed through studies with human samples and rat and rabbit models [31,32,33,34]. Amniotic fluid from CDH pregnancies showed metabolic differences from controls, though their direct link to CDH pathogenesis remains unclear [35]. The authors claim that the metabolomic profile can be used as a biomarker to distinguish CDH amniotic fluid from controls. Postnatal metabolomic studies revealed distinct metabolic profiles in tracheal aspirates of CDH infants [31]. In the nitrofen rat model, NMR-based metabolomics identified disruptions in glycolysis, antioxidants and nucleotide metabolism [34]. In the surgical rabbit model, pathway analysis in CDH + tracheal occlusion showed enrichment in the ubiquinone, tyrosine and terpenoid-quinone biosynthesis pathways, yet these differences were not observed between CDH and sham controls [33]. These metabolic disturbances may contribute to the heterogeneity observed in lung epithelial cells in CDH by influencing epithelial cell differentiation and function, ultimately leading to diverse cellular phenotypes.

The variability in findings across different proteomic and metabolomic studies in CDH may stem from multiple factors, including species-specific differences in lung development, the choice of biological samples (e.g., tracheal aspirates versus amniotic fluid, human vs. animal) and the inherent sensitivity and specificity of different analytical platforms. Furthermore, variations in sample processing, data normalization techniques and the dynamic nature of protein and metabolite expression during lung development may further contribute to these discrepancies, underscoring the need for standardized methodologies and cross-platform validation to ensure reproducibility and biological relevance in CDH research. Despite variability, the aforementioned omics studies have identified common pathways between models, notably regarding inflammation, tight junction stability and growth factor signaling [26,27,28,29]. These pathways have known involvement in epithelial cell function, including epithelial cell differentiation, suggesting that the disruption of these pathways in CDH could underlie epithelial dysfunction [5,11,36]. Findings across proteomic and metabolomic studies regarding CDH are summarized in Table 1. The integration of proteomic and metabolomic approaches has significantly advanced our understanding of the molecular underpinnings underlying CDH pathogenesis, highlighting inflammatory responses and metabolic alterations as central mechanisms and potential therapeutic targets.

### 2.3. Mechanical Disruption as a Driver of Differentiation Defects

Fetal lung development is tightly regulated by mechanical cues, including intra-thoracic pressure, cyclic stretch and lung fluid production. These forces are essential for cell proliferation, branching morphogenesis and lineage-specific epithelial differentiation [37,38,39]. In CDH, the herniation of abdominal contents into the thoracic cavity disrupts these mechanical signals by compressing the developing lungs. The widely accepted dual-hit hypothesis, originally proposed based on observations in the nitrofen rat model, posits that CDH lung hypoplasia results from a primary developmental defect in the lung (first hit) followed by secondary mechanical compression (second hit) [40]. However, growing evidence challenges the necessity of an intrinsic first hit. An alternative model suggests that a perturbation in diaphragm development—potentially analogous to neural tube defects—leads to the herniation of viscera, with subsequent lung compression acting as the primary driver of downstream pulmonary pathology.

Mechanical compression impairs lung epithelial differentiation by disrupting mechanotransduction. Specifically, it reduces nuclear localization of the transcription factor YAP, a central effector of the Hippo pathway and orchestrator of embryonic lung development [4,12,41,42]. The loss of nuclear YAP activity correlates with increased NF-κB signaling in lung epithelial cells, initiating a proinflammatory transcriptional program and impairing epithelial differentiation [4,11]. Notably, this process begins early in gestation: as early as 21 weeks, human CDH lungs exhibit reduced nuclear YAP in epithelial cells [4]. Of note, YAP had previously been implicated in CDH pathogenesis [43].

This mechanistic axis—the loss of nuclear YAP, NF-κB activation, and impaired alveolar differentiation—has been observed consistently across species [4]. In human CDH lungs, an increase in SPC+ ATII cells and a reduction in HOPX+ alveolar type I (ATI) cells indicate disrupted epithelial differentiation. The nitrofen rat model mirrors this phenotype, showing reduced nuclear YAP and expansion of SPC+ populations in CDH offspring but not in nitrofen-treated pups without hernia. In the surgical lamb model of CDH, we demonstrated that YAP inactivation correlates with NF-κB activation and alveolar differentiation defects. Importantly, these abnormalities are reversible: FETO restored nuclear YAP localization, normalized NF-κB levels and rescued alveolar differentiation in CDH lambs. The initiation of inflammation via failed mechanotransduction and the loss of YAP activity due to lung compression might trigger a cascade that recruits additional inflammatory mediators, including macrophages, which have recently been implicated in CDH pathogenesis [10,13]. This may create a self-perpetuating inflammatory environment that further disrupts epithelial integrity and impairs lung development.

Together, these findings suggest that mechanical disruption is not a secondary consequence but a primary pathogenic driver in CDH. Thus, accurately modeling CDH requires replicating the compression defects characteristic of the disease. The consistent pattern of YAP reduction, NF-κB activation and epithelial differentiation failure—observed in human, rat and sheep lungs—positions lung compression as the unifying insult in CDH pathogenesis [4]. While additional genetic or environmental factors may contribute to lung hypoplasia in some cases, mechanical compression is likely the dominant and pathognomonic mechanism underlying epithelial dysfunction in CDH [44]. That these defects can be reversed by FETO in the surgical lamb model of CDH highlights its potential to restore normal lung development [4,14]. Optimizing the timing and duration of FETO and combining it with adjunct therapies targeting inflammation and epithelial differentiation may further enhance its therapeutic efficacy.

## 3. Revisiting and Refining Experimental Models of CDH

### 3.1. New Insights from the Nitrofen Rat Model

The nitrofen rat model has been a foundational tool of CDH research for over two decades. The oral administration of nitrofen to pregnant rats at E9 induces lung hypoplasia in all rat pups and diaphragmatic defects in approximately two-thirds [45]. A key limitation of this model is the early postnatal lethality of affected pups, which prevents the evaluation of survival outcomes and postnatal alveolar development. The use of the model has evolved from a tool mainly used to investigate the histomorphological aspects of CDH or single genes to an in vivo platform for dissecting molecular mechanisms and intercellular interactions driving pulmonary hypoplasia.

As previously outlined, the nitrofen model has been instrumental in identifying inflammation as a central contributor of CDH-associated lung hypoplasia [5,10,26]. Proteomic profiling and sc-RNA-seq have implicated dysregulated proinflammatory signaling and enrichment of inflammatory macrophage populations [10,26]. The nitrofen model has also served as a platform to evaluate therapeutic strategies targeting molecular alterations identified in human CDH. MicroRNAs (miRNAs), small non-coding RNAs that regulate gene expression post-transcriptionally, have emerged as critical regulators of lung development [46,47,48]. Elevated levels of miR-200b in human CDH lungs have been associated with improved neonatal survival [49]. In the nitrofen model, miR-200b is downregulated, and increasing miR-200b abundance rescued lung hypoplasia and reduced the incidence of diaphragmatic defects [50]. This illustrates a translational pipeline moving from bedside to bench, with potential for back-translation to the bedside.

Recent studies have extended the nitrofen model to include lung organoids derived from nitrofen-exposed fetal lungs [7]. These organoids have been used to evaluate regenerative interventions such as extracellular vesicles (EVs) derived from amniotic fluid stem cells (AFSC-EVs). The administration of AFSC-EVs restored epithelial proliferation and promoted differentiation, as evidenced by an increased expression of SPC and CC10. These findings support the utility of nitrofen-derived organoids as a complementary in vitro system for studying CDH pathogenesis and for therapeutic screening.

### 3.2. Air–Liquid Interface Culture Models

One of the most significant advances in CDH research for modeling the human neonatal lung epithelium in recent years has been the use of air–liquid interface (ALI) cultures [5,11]. In this system, patient-derived basal stem cells (BSCs) are seeded on permeable membranes with basolateral contact to media and apical exposure to air, mimicking the in vivo epithelial niche and promoting differentiation into a pseudostratified mucociliary epithelium over three weeks [5,11,51,52,53]. This approach bypasses the need for pluripotent stem cells, enables direct modeling of patient-specific epithelial phenotypes and is both cost-effective and scalable. Importantly, BSCs can be isolated from routinely collected tracheal aspirates from intubated neonates—considered medical waste—allowing for non-invasive sampling with a virtually unlimited supply and high derivation success rates [51]. We have successfully leveraged these patient-specific epithelial cultures for disease modeling, functional assays and therapeutic interrogation in a multitude of lung diseases [5,11,51,53].

Using bulk RNA sequencing, we identified a disease-specific proinflammatory signature in CDH BSCs, characterized by hyperactivated NF-κB signaling and confirmed at the protein level by Western blot [5]. Differentiated ALI cultures from CDH BSCs retained basal cell markers (KRT5+) and exhibited significant deficits in secretory differentiation, particularly of CC10+ club cells, compared to controls. These features were partially recapitulated in human fetopsy lung samples, underscoring the translational fidelity of the in vitro model. Corticosteroid treatment with dexamethasone of CDH BSCs reversed NF-κB hyperactivation and restored secretory differentiation, providing a proof of concept for pharmacologic rescue and clinical translation. These findings were supported by in vivo experiments in the nitrofen rat model, where prenatal administration of dexamethasone and the NF-κB inhibitor JSH-23 demonstrated similar effects. Of note, in humans, the prenatal use of steroids in CDH was evaluated in a randomized controlled trial aimed at enhancing surfactant production and improving pulmonary maturation [54]. Although no clinical benefit was observed, this may be attributed to factors such as the timing, dosage, or route of administration (administration via the mother). Additionally, the heterogeneity of CDH suggests that steroids alone may be insufficient or ineffective in some cases, and the small sample size (34 patients) likely limited the statistical power of the study [54]. Additionally, we demonstrated that BSCs serve as a powerful platform for epigenetic profiling via ATAC-sequencing. Chromatin accessibility in CDH BSCs was profoundly altered, with significant enrichment of binding motifs for NF-κB and AP-1 family members, suggesting priming toward inflammatory activation [5]. We found increased signal density near the transcription start sites of key inflammatory and differentiation genes, including *MYD88*, *IRF2* and *TGFB1*. The ability to perform high-resolution chromatin profiling directly on primary human airway progenitors highlights the versatility of this model and offers a framework for uncovering regulatory defects in other neonatal lung diseases.

To further dissect the molecular drivers of epithelial differentiation defects in CDH, we investigated the intersection of mechanical and inflammatory signaling. Our recent work revealed that CDH BSCs exhibit dysregulated YAP signaling, correlating with elevated NF-κB activity and impaired epithelial differentiation [11]. Pharmacologic restoration of YAP signaling via LATS kinase inhibition normalized NF-κB activity and rescued differentiation defects, implicating disrupted mechanosensing due to in utero lung compression as a central driver of inflammation and impaired epithelial differentiation in CDH.

Taken together, the integration of patient-derived BSC and ALI models, transcriptomic and epigenomic profiling and mechanistic perturbation studies has deepened our understanding of CDH lung pathogenesis and established a platform for therapeutic targeting.

### 3.3. Organoid Culture for Modeling the CDH Lung

Organoids are three-dimensional structures derived from stem/progenitor cells that recapitulate developmental, functional and structural aspects of their in vivo counterparts, making them advantageous over two-dimensional models to investigate biological processes and therapeutic testing. Although organoids were first developed using mouse intestinal stem cells to model the gut, they are now widely used to model a variety of organs and tissues in vitro, including the lung [55,56,57,58,59,60,61,62].

Organoids hold several advantages over 2D ALI cultures. While ALI cultures, as discussed, can model the developed, proximal airway epithelium, organoids enable the modeling of earlier developmental events such as lung progenitor specification and branching morphogenesis, as well as proximal/distal epithelial differentiation [4,5,58,59,63,64]. Furthermore, unlike ALI cultures, organoids are capable of recapitulating the alveolar niche [59,65,66,67,68]. ‘Alveolospheres’ can be created from AT2 cells, which differentiate to AT1s in culture [69]. Fetal stem cell-derived bud tip progenitor cells can be differentiated to create either airway or alveolar organoids, while iPSC-derived patient-specific cells can be differentiated to recapitulate developmental processes and result in airway, alveolar, ‘lung’ or bud tip organoids [58]. Patient-derived organoids offer a unique opportunity to investigate disease mechanisms and enable patient-specific therapeutic testing [58,61]. Additionally, organoids can contain different cell types and be used to investigate cell–cell interactions. Established protocols allow the generation of organoids containing both epithelial and mesenchymal cell lineages, and epithelial and immune cell co-culture models exist, such as macrophage-containing alveolar organoids [60,70,71]. One limitation of organoids in modeling the in vivo phenotype is their lack of vascularization, which has currently only been achieved through transplantation into host species [59,70]. Furthermore, airway organoids often organize in an inward orientation, which can affect their ability to respond to airborne stimuli and limit their use in compound testing [72]. Despite these limitations, organoids have emerged as important models to model diseases and have been able to advance many areas of biological lung research, including CDH.

CDH affects multiple cell types at different developmental stages. Organoids offer a promising platform to recapitulate disease phenotypes, though their application in CDH research is in its infancy. The recognition of the need to manipulate mechanical compression during differentiation, as would occur in in vivo CDH lung development, has resulted in attempts to create mouse lung CDH organoid models [73]. Either mediated by forskolin or cyclic strain, mouse CD326+ve cells and lung fibroblasts were subjected to compression during organoid culture. Disrupting biomechanics altered the development and differentiation of the organoids, and while not fully recapitulating CDH phenotypes, an increase in basal and AT1/AT2 progenitor cells was observed, as seen in other CDH models [4,5,73]. Changes in gene expression of co-cultured fibroblasts were also identified, illustrating the importance of compression-mediated epithelial–mesenchymal interactions in CDH [73].

The importance of altering compression in vitro to mimic CDH in lung organoid culture was also identified by others [74]. Using iPSC cells derived from patient-specific amniotic fluid somatic cells, ‘lung-like’ organoids were differentiated with or without compression [74]. CDH patient-derived organoids showed impaired generation of NKX2.1^+^ progenitors, type II alveolar epithelial cells and PDGFRα^+^ myofibroblasts. On compression, these organoids exhibited decreases in *SOX2* and *SOX9* gene expression and a downregulation of *PDPN* and *NKX2.1*, illustrating a potential loss of AT1 cells, as seen in CDH models [4,74]. Success in recapitulating CDH phenotypes in organoid models was partially achieved by directly differentiating lung organoids from CDH fetal amniotic fluid cells or fetal tracheal cells [5,14]. These organoids showed decreased club and basal cells, increased neuroendocrine cells and an upregulation of surfactant protein genes, illustrating an increased presence of AT2 cells, although a manipulation of biomechanical forces was not conducted [14]. Combining this approach with compression could refine the development of patient-specific disease models that faithfully mimic epithelial and alveolar phenotypes and cell–cell interactions.

### 3.4. Ex Vivo Lung Explants

Ex vivo lung explants serve as effective CDH models, as they retain native tissue architecture. Unlike organoids, ex vivo explants retain vascular integrity and, crucially, contain a variety of immune cells [75,76,77]. The diverse range of cell types found in ex vivo explants allows for the study of epithelial–stromal, epithelial–immune and various other cell–cell interactions, which has become increasingly important in CDH research since the discovery of an aberrant inflammatory phenotype [4,5]. Furthermore, lung explants are well suited for modeling lung compression.

In a recent study, whole nitrofen-exposed fetal rat lungs were explanted and subjected to mechanical compression with pressure settings mimicking in vivo abdominal organ herniation [78]. This model, specifically designed to investigate the role of changes in mechanical forces on lung development, found that compression significantly impaired lung development, both transcriptionally and morphologically. Interestingly, both control and nitrofen-treated rats exhibited similar epithelial differentiation defects, suggesting that compression is a primary driver of epithelial cellular defects in CDH. On the other hand, the upregulation of *ACTA2* was only seen in nitrofen- and compression-exposed fetal explants, consistent with previous findings of mesenchymal tissue defects in CDH [79]. Another study examined the therapeutic efficacy of VEGF—previously found to be downregulated in CDH models—in a lung explant compression model [80]. Similarly, compression alone was sufficient to induce structural and transcriptional changes consistent with lung hypoplasia. VEGF treatment reversed key features of CDH-associated pulmonary hypoplasia, and the subsequent application of VEGF in the in vivo nitrofen rat model recovered lung growth and pulmonary arterial remodeling, illustrating the value of ex vivo models for disease modeling and therapeutic testing [80].

Other groups have attempted to model the effect of tracheal occlusion (TO) using explant cultures. Nitrofen-treated rat lungs were explanted and subjected to TO in culture. Analysis of lung molecular markers after three days evidenced a decrease in SPC proteins in TO lungs, in conflict with previous findings [4,81]. This may be attributed to an increased abundance of caspase-3-positive cells, likely resulting from limited nutrient absorption after three days in culture. Taken together, this highlights the usefulness of multicellular models and positions ex vivo lung explants as a promising area of future research.

Another example of ex vivo models is precision cut lung slices (PCLS). PCLS are capable of alveolarization ex vivo and allow for advanced imaging and compound testing [82,83]. While theoretically ideal as they retain full tissue architecture, the unique structure of the lung as an ‘air filled’ space requires the replacement of air with low-melting-point agarose prior to slicing [82,83]. This creates a compressed environment within the lung, which, as extensively discussed in this review, can affect many aspects of cellular processes in the lung. Nevertheless, with proper controls, this technique can be very useful to compare diseased vs. control lungs and could be utilized for CDH research due to it being one of the few models able to recapitulate vascularization.

While emerging models have advanced our understanding of CDH lung pathogenesis, discrepancies between the findings—particularly regarding epithelial differentiation and the effects of interventions such as FETO—highlight the need for standardized methodologies. Differences in species, developmental stage, culture conditions and compression regimens may contribute to these inconsistencies. Cross-species validation and the establishment of reproducible, physiologically relevant compression protocols could help resolve these conflicts. Future studies should prioritize model standardization and integrate comparative analyses across organoids, explants and in vivo systems to strengthen translational relevance and reproducibility.

## 4. Novel Therapeutic Strategies Targeting Epithelial Dysfunction

### 4.1. Stem Cell and Regenerative Therapies

A growing body of work has explored the potential of regenerative and stem cell-based therapies to improve lung development in CDH. AFSC-EVs are nano-sized vesicles secreted by pluripotent stem cells isolated from amniotic fluid. These vesicles carry a bioactive cargo, including regulatory miRNAs, mRNAs and proteins, that modulate key developmental and inflammatory pathways in recipient cells [7,84]. In the nitrofen rat model, AFSC-EVs restored the expression of autophagy activators suppressed in nitrofen-exposed lungs and improved branching morphogenesis in lung explants from the pseudoglandular and canalicular stages. In vivo, intra-amniotic administration at E21 (canalicular stage) rescued impaired autophagy, a process linked to the miR-17~92 cluster [85]. AFSC-EVs also promoted epithelial maturation in ex vivo and in vivo CDH rat lungs, as evidenced by increased expression of SPC and CC10, and enhanced branching morphogenesis at canalicular and saccular stages [10,86]. Additionally, in mesenchymal cultures derived from nitrofen-exposed lungs, AFSC-EVs improved PDGFRA and lipofibroblast marker expression, suggesting enhanced mesenchymal maturation [86]. Beyond epithelial and autophagy rescue, AFSC-EV reduced proinflammatory macrophage infiltration in CDH rat lungs, indicating broader immunomodulatory activity [10]. These findings position AFSC-EVs as a promising regenerative therapy for correcting epithelial defects in CDH lungs while modulating the inflammatory and mesenchymal microenvironment.

Mesenchymal stem cell-derived extracellular vesicles (MSC-EVs) represent another stem cell-based therapeutic strategy. While extensively studied in bronchopulmonary dysplasia and acute respiratory distress syndrome models, their application in CDH remains limited [87,88,89]. In the nitrofen rat model, intravenous MSC-EVs administration at birth attenuated pathological extracellular matrix remodeling in the pulmonary vasculature [90]. Treatment inhibited ECM-modifying enzymes, including LOX and MMP-9, and partially restored vascular structural integrity [90]. Although epithelial effects were not assessed, these findings support MSC-EVs as a potential strategy to mitigate CDH-associated pulmonary hypertension.

Lastly, transamniotic stem cell therapy (TRASCET) involves the intra-amniotic administration of stem cells, most commonly amniotic fluid-derived mesenchymal stem cells (afMSC) [91,92]. This approach aims to enhance fetal repair mechanisms by allowing donor cells to be absorbed via fetal swallowing and breathing, distributing cells systemically and to injury sites. In the nitrofen model, TRASCET ameliorated both pulmonary vasculature and epithelial development. AfMSC administration reduced arteriole wall thickness, downregulated endothelial nitric oxide synthase and endothelin receptor-A expression and improved pulmonary vascular resistance [93,94]. Donor cells were detected in bone marrow and umbilical cord, indicating systemic distribution [91]. Another study reported increased expression of SPC and reduced levels of FGF-10 and VEGF-A in treated lungs, suggesting partial rescue of epithelial differentiation with altered growth factor signaling [93]. Although mechanistic insights are limited and evidence is confined to the nitrofen model, these findings support stem cell therapies as a minimally invasive, biologically compatible approach to modulating lung development in CDH.

### 4.2. Future Directions: Precision Medicine and Defining CDH Endotypes

CDH is a multifactorial condition, characterized by a diaphragmatic defect resulting in utero lung compression, and is influenced by genetic and environmental factors. This is illustrated by its sporadic nature, the small number of familial cases and the heterogeneity of clinical presentation of CDH patients [23,95]. A study analyzed 218 CDH-associated genes and conducted gene ontology enrichment analysis to identify causative pathways of varying CDH phenotypes [95]. Anatomical subtypes of CDH, such as Bochdalek hernia, eventration or central tendon defects, were each associated with unique sets of genes and biological processes—including retinoic acid signaling, myogenesis and angiogenesis, respectively [80]. Their findings suggest that different forms of CDH arise from divergent molecular mechanisms, reinforcing the concept of disease heterogeneity. The heterogeneity of CDH has also been seen in vitro. Studies using patient-derived BSCs have revealed variable epithelial dysfunction, with only a subset of BSCs recovering their differentiation capacity after anti-inflammatory treatment with dexamethasone [5]. As discussed in this review, lung compression alone is sufficient to induce key features of the CDH phenotype [3,5,11]. While some patients may primarily be affected by mechanical lung compression, others may exhibit additional molecular or epigenetic abnormalities that exacerbate disease severity [5]. In line with this, endotype classification could reflect varying responses to therapies. A ‘compression-only’-induced inflammatory phenotype, for example, may respond differently to anti-inflammatory steroidal options than more complicated phenotypes, which may require additional therapies. The findings from the aforementioned research investigating novel therapeutic strategies and their outcomes are summarized in Table 2.

Defining distinct CDH endotypes through transcriptomic profiling and biomarker discovery is essential for the development of future therapeutic strategies and the stratification of disease severity. The relevance of patient-specific biomarkers is exemplified by miR-200b expression in tracheal fluid collected during FETO balloon removal, which correlates with improved outcomes, likely due to the miR-200b-mediated suppression of TGFβ-SMAD signaling [49,96]. Conversely, elevated *GATA4* expression, encoding a transcription factor critical in diaphragm and lung development, has been reported in some CDH patients and may serve as a genetic biomarker for more severe phenotypes [8]. Overall, future studies on patient-derived lung cell models (e.g., BSCs, organoids) and transcriptomic analysis may reveal common pathways and allow for classification in different disease endotypes, thereby enabling targeted (prenatal) therapies.

## 5. Conclusions

Advances in transcriptomic, proteomic, and in vitro modeling approaches have transformed our understanding of epithelial dysfunction in CDH. A growing body of evidence supports mechanical compression as a central pathogenic driver, acting through dysregulated mechanotransduction and inflammatory signaling to impair epithelial differentiation. Integrating patient-derived models with high-resolution molecular profiling holds promise for identifying additional patient-specific CDH endotypes, enabling personalized therapeutic strategies to restore normal lung development.

## Figures and Tables

**Table 1:** Comparison of findings from proteomic and metabolomic studies across CDH models.

Model (Developmental Stage + Sample Type)		Omics Method	Findings	Ref.
**Nitrofen Rat**	Canalicular; lung tissue	LS-MS/MS	>200 altered proteins, inflammatory response.	[25]
	Pseudoglandular; lung tissue	Tandem mass tag (TMT)	79 altered proteins. Tight junction pathways, phospholipase D pathways and HIF-1 signaling.	[26]
	Late gestation; lung tissue	NMR-based metabolomics	Disruption in glycolysis, nucleotide metabolism, antioxidants.	[33]
**Human**	Canalicular and alveolar; amniotic fluid	Proteomics + ELISA validation	218 altered proteins. GP6 signaling. MSP-RON signaling. Cardiovascular development.	[28]
	Alveolar; umbilical cord serum	LC-MS/MS	98 altered proteins. Complement C1q/C5. Coagulation cascades.	[29]
	Canalicular, saccular and alveolar; amniotic fluid and tracheal aspirates	Metabolomics	Distinct CDH metabolomic profiles.	[34]
**Lamb** (surgical)	Saccular; tracheal fluid	Proteomics	CDH suppressed (while TO promoted), cell proliferation and AKT-related signaling cascades.	[27]
**Rabbit** (surgical)	Late saccular; lung tissue	Metabolics + pathway analysis	Enrichment in ubiquinone, tyrosine, and terpenoid-quinone biosynthesis in CDH + TO.	[32]

**Table 2:** Summary of novel therapeutic strategies targeting epithelial dysfunction in CDH. Stem cell and regenerative therapies were all tested in the nitrofen rat model.

Approach		Target	Outcome	Ref.
**Stem cell and Regenerative Therapies**	AFSC-EVs	Developmental and inflammatory pathways	Autophagy activator expression restored. Promoted branching morphogenesis. Promoted epithelial and mesenchymal maturation. Reduced inflammation.	[82,83]
	MSC-EVs	ECM-modifying enzymes	Partial restoration of vascular structural integrity.	[87]
	TRASCET	Pulmonary vasculature and epithelial development	Improvement of epithelial differentiation and vascular development.	[90,91]
**Precision Medicine and CDH Endotyping**	Transcriptomic profiling	Biomarker identification	miR-200b expression correlates with improved outcomes. Elevated *GATA4* marks more severe phenotypes.	[8,93]
**Anti-NFkB Signaling**	DEX and JSH-23	Reduction of inflammation	Benefit seen in rat model, though not in human trials (discussed in Section 3.2).	[53]

## Data Availability

No new data were created or analyzed in this study.
